# Reevaluating 30 cmH_2_O endotracheal tube cuff pressure: risks of airway mucosal damage during prolonged mechanical ventilation

**DOI:** 10.3389/fmed.2024.1468310

**Published:** 2024-11-25

**Authors:** Guo Mu, Feixiang Wang, Qiang Li, Xuan Yu, Bin Lu

**Affiliations:** ^1^Department of Anesthesiology, Zigong Fourth People’s Hospital, Zigong, China; ^2^Anesthesiology and Critical Care Medicine Key Laboratory of Luzhou, Southwest Medical University, Luzhou, China

**Keywords:** airway, endotracheal tube, cuff pressure, endotracheal mucosa, threshold

## Abstract

**Background:**

The optimal endotracheal tube (ETT) cuff pressure remains contentious. In the traditional consideration that the level 30 cmH_2_O is considered safe, balancing the prevention of reflux aspiration against airway mucosal damage. Whether this pressure level can cause potential damage to the airway mucosa remains to be discussed.

**Methods:**

Airway mucosa damage and structural changes at 30 cmH_2_O were examined in patients under general anesthesia and in rabbit mechanical ventilation models. Prior to this, we also interviewed some anesthesiologists about the level of concern about ETT cuff pressure.

**Results:**

A total of 634 valid questionnaires suggested that anesthesiologists generally do not pay enough attention to ETT cuff pressure and the average established cuff pressure significantly exceeded 30 cmH_2_O. Airway mucosa images of 100 general anesthesia patients with different ventilation duration indicated that maintaining the pressure at 30 cmH_2_O did not cause significant damage to airway mucosa in a short period of time, while it still caused damage to airway mucosa in patients with long-term ventilation, with damage severity increasing with longer ventilation periods. This correlated strongly with postoperative sore throat (*R*^2^ = 0.3884, *p* < 0.001). In rabbits, 4 h of ventilation at this pressure resulted in significant loss of ciliated epithelium and inflammation. Calculations suggested an effective dose (ED_50_) to prevent mucosal injury at a cuff pressure of 25.64 cmH_2_O (95% CI: 19.268–29.367 cmH_2_O).

**Conclusion:**

The currently established cuff pressure of 30 cmH_2_O is associated with airway mucosal damage in both clinical and animal models. Lowering the safety threshold of cuff pressure may be necessary to mitigate mucosal injury.

## Introduction

Endotracheal intubation is indispensable in general anesthesia, pre-hospital emergency care, and intensive care units for airway control. The endotracheal tube (ETT) cuff, a crucial component, not only maintains airway seal for assisted ventilation but also effectively prevents aspiration (1). However, establishing appropriate ETT cuff pressure poses challenges for anesthesiologists. Insufficient pressure increases the risk of ventilator-associated leaks and fails to prevent leakage of oropharyngeal secretions into the lower airways, closely linked to ventilator-associated pneumonia (VAP) (2). Conversely, excessively high cuff pressures pose significant patient risks. Even brief periods of elevated cuff pressure can lead to tracheal mucosal damage, including ischemia, ulcers, necrosis, tracheoesophageal fistula, and potentially fatal tracheal rupture (3, 4). The safety threshold for ETT cuff pressure remains contentious; currently, 30 cmH_2_O is considered safe, balancing prevention of aspiration against airway mucosal damage (5–7). However, perioperative factors frequently alter cuff pressures, and comprehensive data on the microstructural effects of 30 cmH_2_O cuff pressure on tracheal mucosa are lacking (8). Reevaluating 30 cmH_2_O as a safe threshold to prevent tracheal mucosal injury may be necessary.

**Figure 1:**
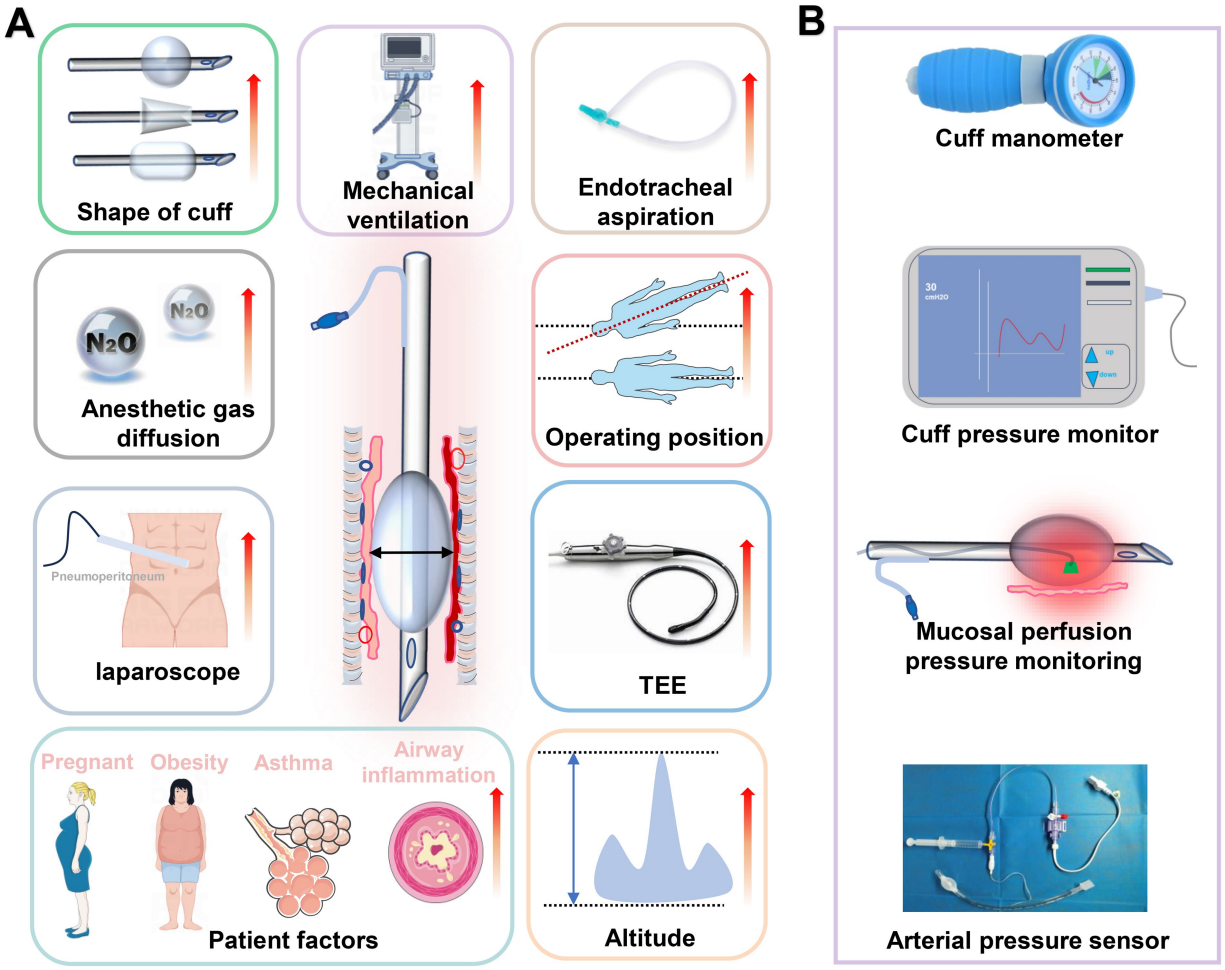
Review of risk factors and monitoring methods for increasing ETT cuff pressure. **(A)** Summary of the main risk factors for increasing ETT cuff pressure. **(B)** Tools to monitor ETT cuff pressure.

The tracheal mucosa is extremely fragile. The hydrostatic pressure of the tracheal mucosal capillaries is approximately 24 cmH_2_O in adults, and that of the lymphatic vessels is around 7 cmH_2_O. When the internal pressure of the cuff exceeds 35 cmH_2_O, the blood flow at the compressed section of the trachea significantly decreases, increasing the risk of tracheal injury (3). It is widely accepted that maintaining cuff pressure at or below 30 cmH_2_O is crucial to prevent damage to the tracheal mucosa. However, current attention to ETT cuff pressure among anesthesiologists is inadequate, with numerous surveys indicating that the pressures set frequently exceed this safe threshold (9, 10). Moreover, various factors in clinical practice, such as the specifics of anesthesia and surgical procedures, patient-specific airway characteristics, and even altitude, can significantly elevate cuff pressure (11–19) (Figure 1A). Therefore, monitoring and appropriately adjusting cuff pressure is essential for the protection of the tracheal mucosa. Tools such as manometers, continuous cuff pressure monitoring and adjustment devices, mucosal blood flow monitors, and standard clinical pressure sensors support the careful monitoring and adjustment of cuff pressure (3, 20, 21) (Figure 1B). Nevertheless, there is a lack of clear threshold standards for cuff pressure, and a critical issue that demands close examination is the reference levels for cuff pressure adjustments, aimed at maximizing the prevention of VAP and tracheal mucosal damage.

It is generally believed that maintaining cuff pressure between 20–30 cmH_2_O is appropriate for adult patients under normal circumstances (5). Pressures below 20 cmH_2_O are independently associated with an increased risk of VAP, while 30 cmH_2_O serves as a safe upper limit to prevent tracheal mucosal injury, and is currently a primary reference for adjusting cuff pressure (2). However, the safety margins regarding cuff pressure are based on theoretical deductions about mucosal perfusion pressure, and there is a lack of evidence to confirm whether 30 cmH_2_O as a reference cuff pressure could cause damage to the mucosa in patients under prolonged general anesthesia. There is also a dearth of research on whether 30 cmH_2_O could potentially harm the microstructure of the tracheal mucosa. This study aims to reassess the safety limits for cuff pressure to prevent tracheal mucosal damage through current status surveys, clinical imaging, and mechanical ventilation animal models.

## Methods

This study was conducted as a multifaceted study comprising three parts. Ethical approvals were obtained from the Ethics Committee of the Zigong Fourth People’s Hospital (ID: 2022-090). In brief, the first part of the study utilized a web-based questionnaire survey among anesthesiologists primarily in Sichuan Province, China, to assess their methods and awareness regarding the establishment of ETT cuff pressures across different proficiency levels. Additionally, pressure monitoring was used to measure ETT cuff pressure values established by select anesthesiologists, and the impact of perioperative anesthesia and surgical procedures on cuff pressures was evaluated. The second part of the study involved a retrospective observational analysis of the condition of airway mucosa in mechanically ventilated general anesthesia patients maintained at a baseline ETT cuff pressure of 30 cmH_2_O for varying durations. Correlation analyses were performed between the degree of mucosal damage and postoperative pharyngeal pain severity based on postoperative follow-up records. In the third part, an animal mechanical ventilation model was employed to maintain ETT cuff pressure at 30 cmH_2_O. After 4 h of ventilation, microscopic structural changes in the airway mucosa were observed, and the sequential method was used to estimate the ED_50_ of cuff pressure required to prevent airway mucosal injury.

### Questionnaire survey

The survey component of this study was conducted using an online questionnaire platform (Wenjuanxing, www.wjx.cn), targeting anesthesiologists of various professional ranks in the Sichuan region of China at random. The questionnaire primarily focused on aspects such as methods employed by anesthesiologists to inflate the endotracheal tube (ETT) cuff, the importance attributed to this process, and the techniques used to monitor cuff pressure. The questionnaires were delivered in the form of the research team contacted the heads of different anesthesia departments in advance, and the heads of the departments sent network links within the departments. In order to obtain more comprehensive information, we did not limit the title and working years of anesthesiologists who participated in the questionnaire, but non-anesthesiologists were excluded. Questionnaires that did not undergo complete information entry were excluded. In order to avoid participants filling in the questionnaire at will, we also excluded questionnaires with an overall questionnaire response time of less than 3 min.

### ETT cuff pressure measurement

The research team randomly measured ETT cuff pressure actually established by some of the anesthesiologists who responded to the questionnaire. After endotracheal intubation was completed in adult patients, the cuff pressure was measured by connecting the barometer (CPA-A, Kangle Medical Technology Co., Ltd., China) to the ETT guide balloon, the patients with the first elective operation in each operating room were selected for measurement. The cuff pressure of the endotracheal tube was measured using a hand-held barometer within 30 min of the completion of intubation. The general data of patients, surgical and anesthesia parameters, and the information of anesthesia insufflators were recorded. After measurement, the cuff pressure was adjusted to the recommended range (20 to 30 cmH_2_O) and could not be reported to the physician performing the cuff inflation.

### Retrospective analysis

This retrospective analysis utilized the patient database established by the Department of Anesthesiology at Zigong Fourth People’s Hospital, focusing on patients who underwent elective surgeries under general anesthesia with endotracheal intubation. Prior to surgery, the cuff pressure of the ETT was adjusted to 30 cmH_2_O using a barometer. In some patients, a fiberoptic bronchoscope was inserted past the vocal cords to the tip of the ETT during anesthesia to capture preoperative images. At the end of the surgery, the cuff was deflated, and the ETT was partially withdrawn under the direct vision of the bronchoscope to assess the tracheal mucosa at the cuff contact sites.

Inclusion criteria encompassed all adults over 16 years old, with recorded ETT cuff pressure maintained at 30 cmH_2_O, from January 2022 to April 2024. Exclusion criteria included patients from whom preoperative and postoperative tracheal mucosal images could not be obtained, those whose image quality was not clear enough to assess the mucosal condition were also excluded, those unable to report postoperative sore throat, patients with concomitant respiratory diseases, those with pharyngeal or laryngeal conditions, and patients who could not be extubated postoperatively. Data collection involved a retrospective analysis of the selected patients, categorized into cohorts based on the duration between intubation and extubation (2, 4, 6, 8, 10 h). The extent of postoperative airway mucosal damage was scored as follows: 0 point for no injury, 1 for pinpoint congestion, 2 for patchy congestion, 3 for mucosal disruption with bleeding, 4 for mucosal ulceration, and 5 for tracheal perforation. An analysis correlating the degree of mucosal injury with postoperative sore throat was performed.

### Rabbit mechanical ventilation model

A total of 35 adult male New Zealand rabbits, weighing 2–2.3 kg, were procured from Beijing Huafukang Bioscience Co., Ltd. (No. 510137000220002372). The housing conditions were maintained at a temperature of 20–25°C with a humidity of 45–65%. A 12:12 h light-dark cycle was observed. Animals were housed individually and acclimatized for 5 days with *ad libitum* access to water and feed, which were regularly replenished by dedicated personnel. Intramuscular injections of 20 mg/kg ketamine and 0.5 mg/kg midazolam were administered to achieve anesthesia. After onset of anesthesia, an auricular vein was cannulated for infusion of propofol at a rate of 30 mg/kg/h to maintain anesthesia, supplemented with rocuronium bromide at 0.6 mg/kg. An ID 3.5 cm standard ETT was inserted into the trachea until positioned approximately 10 cm from the incisors. The ETT cuff pressure was adjusted to 25, 30, or 45 cmH_2_O, and mechanical ventilation was initiated to maintain temperature and fluid stability. After 4 h of mechanical ventilation, the ETT cuff was deflated. Following a 30 min stabilization period, rabbits were euthanized injection of sodium pentobarbital (100 mg/kg) under adequate anesthetic sedation, and tracheal tissues were harvested. The pathological observation methods of tracheal mucosa included transmission electron microscopy (TEM, HITACHI- ht7800, Japan), hematoxylin and eosin (H&E) staining and terminal deoxynucleotidyl transferase-mediated dUTP-biotin nick end labeling assay (TUNEL) staining.

### Determination of minimum safe cuff pressure

Histopathological examination of the tracheal tissue from the cuff compression area was conducted. Under light microscopy, damage to the mucosa was defined as a “positive” pressure test result, whereas its absence indicated a “negative” pressure test result. The initial pressure inside the tracheal tube cuff was set at 40 cmH_2_O, with adjustments made by increments or decrements of 5 cmH_2_O. A sequential approach was used to determine the cuff pressure for subsequent rabbits based on the results of the previous animal. If a positive test result was observed, the cuff pressure for the next rabbit was reduced by 5 cmH_2_O; conversely, if a negative result was obtained, the pressure was increased by 5 cmH_2_O. The experiment was terminated after observing six transitions from negative to positive outcomes.

### Statistical analysis

All data were statistically analyzed using SPSS software version 28.0 (IBM Corp., Armonk, NY, United States). The normality of the data distribution was assessed using the Kolmogorov–Smirnov test. Normally distributed quantitative data were presented as mean ± standard deviation. Non-normally distributed data were expressed as median and interquartile range. Comparisons among multiple groups were conducted using one-way ANOVA or the Kruskal–Wallis test. The association between mucosal damage and postoperative sore throat was analyzed using linear regression. Pearson correlation analysis was used to analyze the correlation between mucosal injury score and sore throat in retrospective analysis. The minimum safe cuff pressure and its 50% effective dose (ED_50_) and 95% effective dose (ED_95_), along with their 95% confidence intervals (95% CI), were estimated using probit regression analysis. Statistical significance was defined as a two-sided *p*-value of less than 0.05.

## Results

**Figure 2:**
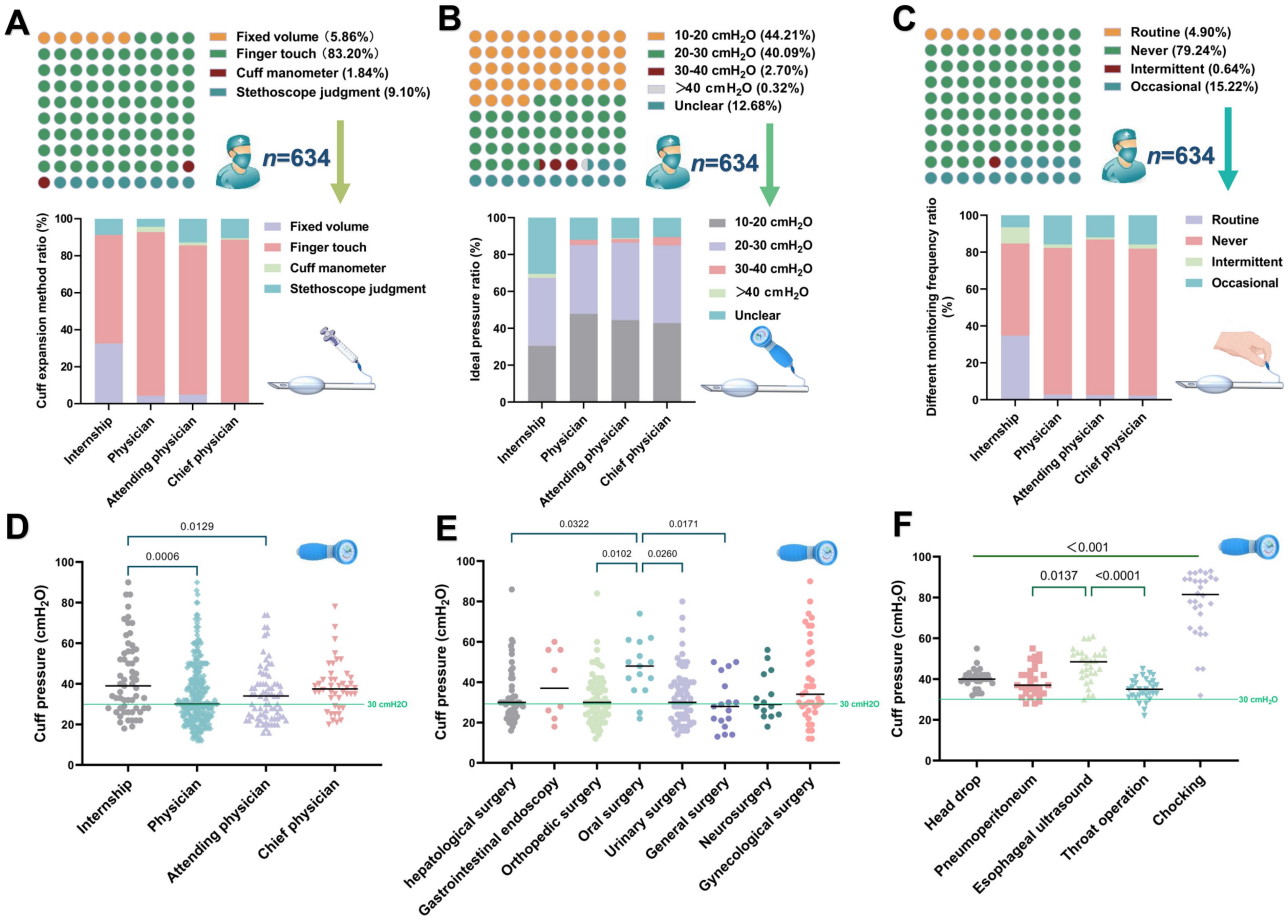
Part of the anesthesiologist establishes a ETT cuff pressure situation. **(A)** Based on questionnaire survey, anesthesiologists used different methods and proportion of ETT cuff expansion. **(B)** The optimal ETT cuff pressure range for different grades of anesthesiologists. **(C)** Different grades of anesthesiologists monitor and adjust the frequency and proportion of ETT cuff pressure. **(D)** Actual ETT cuff pressure values established by anesthesiologists of different professional grades were sampled. **(E)** ETT cuff pressure values established by the anesthesiologist are analyzed twice and ETT cuff pressures established by the anesthesiologist for different types of surgery. **(F)** Effect of perioperative anesthesia and surgical procedures on ETT cuff pressure.

A total of 634 valid questionnaires were obtained. The current method of establishing ETT cuff pressure by most anesthesiologists is incorrect, and pressure monitoring equipment is also lacking. Among these, only 1.84% of anesthesiologists inflated the cuff under the guidance of pressure monitoring devices. The predominant method still employed is the finger touch technique, with its usage frequency increasing progressively as professional titles advance (Figure 2A). In addition, most anesthesiologists have a poor understanding of ETT cuff pressure. There appears to be varying knowledge among surveyed anesthesiologists regarding the appropriate cuff pressure; 44.1% believe that a pressure range of 10–20 cm H_2_O is most suitable, despite such pressure being insufficient to maintain airway seal integrity. Furthermore, 12.86% of anesthesiologists are unaware of the recommended cuff pressure range (Figure 2B). Alarmingly, 79.2% of the anesthesiologists surveyed never monitor or adjust cuff pressure intraoperatively (Figure 2C). The full questionnaire information and results are presented in Supplementary material S1.

The cuff pressures established by 294 anesthesiologists during elective surgeries was measured, and found that the average cuff pressure exceeded safe limits using 30 cmH_2_O as a reference. The highest cuff pressure established by resident physicians was 42.98 ± 18.57 cmH_2_O (95% CI of mean: 38.14 to 47.82 cmH_2_O). We did not observe a decrease in cuff pressure with increasing professional degree (Figure 2D). Secondary analysis of pressure values across different surgical categories revealed significantly higher cuff pressures in oral surgery patients compared to other types of surgeries (Figure 2E). After initial pressure measurements, adjustments were uniformly made to 30 cmH_2_O, and we examined the effects of various anesthesia and surgical procedures on cuff pressure. Coughing resulted in the most pronounced increase in cuff pressure at 77.7 ± 16.68 cmH_2_O (95% CI of mean: 71.47 to 83.93 cmH_2_O), with multiple anesthesia and surgical procedures significantly elevating cuff pressures (Figure 2F).

**Figure 3:**
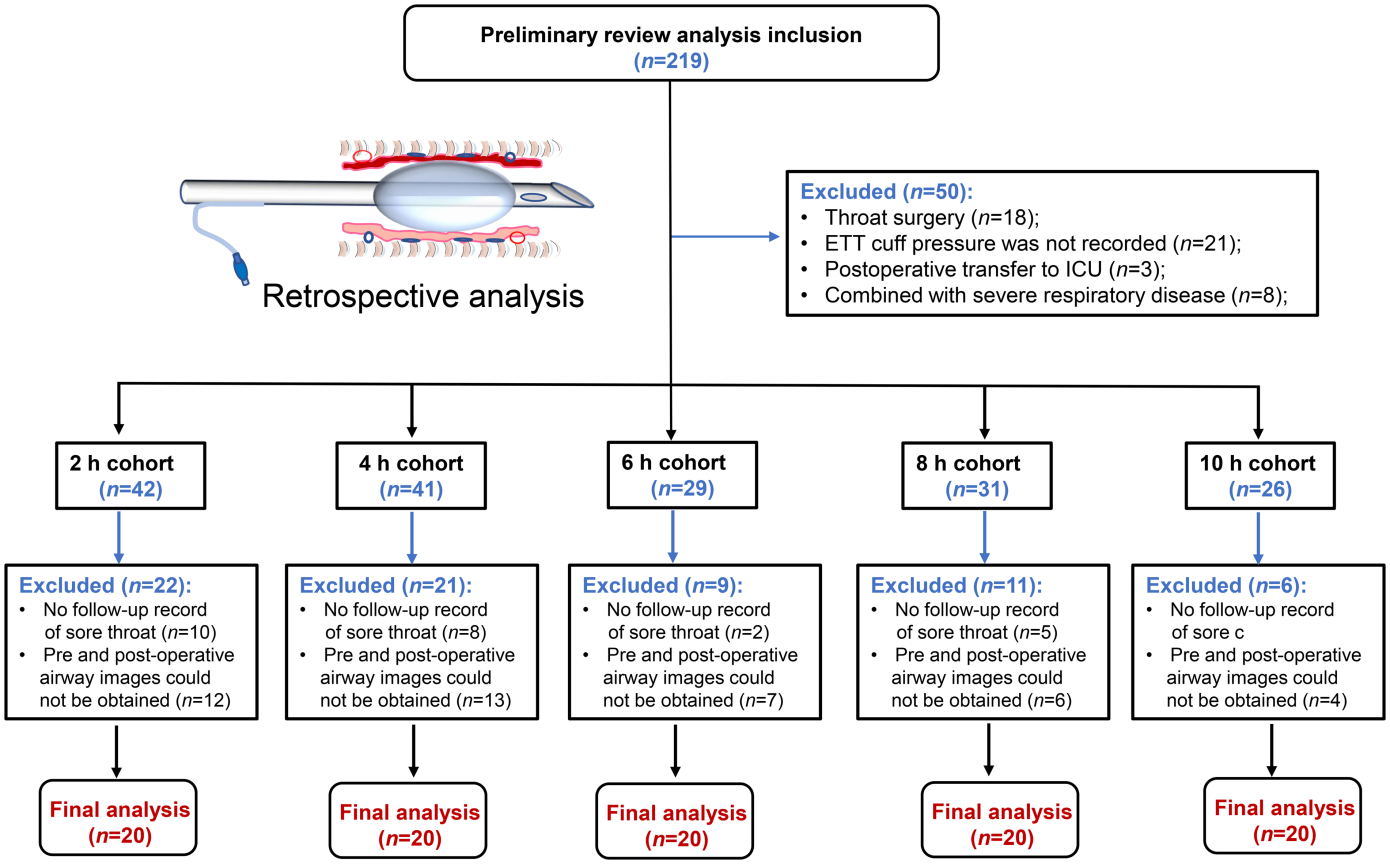
The patients screening flowchart.

**Figure 4:**
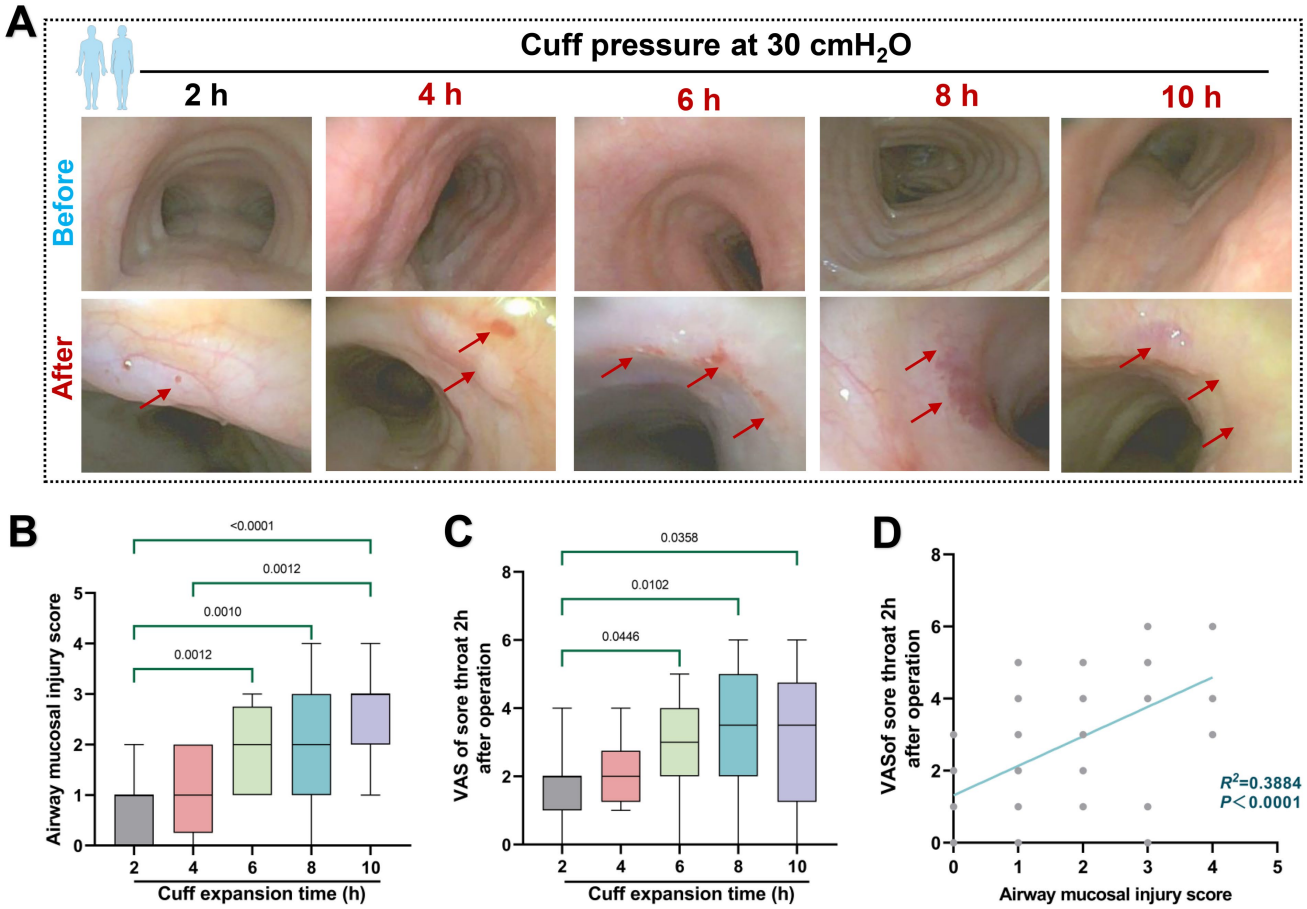
Effect of 30 cmH_2_O ETT cuff pressure on airway mucosa. **(A)** Typical pre- and post-operative images of the effects of ETT cuff pressure set to 30 cmH_2_O on airway mucosa in patients with mechanical ventilation of varying duration. Red arrows indicate changes in mucosal damage. **(B)** Mucosal injury score. **(C)** Retrospective analysis of sore throat VAS scores of patients at 2 h after surgery was included. **(D)** Correlation between mucosal injury score and postoperative sore throat, *R*^2^ = 0.3884.

In the retrospective analysis, 119 of the 219 patients initially screened for inclusion in the study were excluded based on exclusion criteria. Therefore, a total of 100 patients were included in this retrospective study, and complete clinical data were obtained. The flowchart of the patient screening process is shown in Figure 3. At each time point, a total of patients who met the criteria were included in the cohorts. Demographic characteristics of enrolled patients refer to Table 1. For patients ventilated for less than 4 h, a cuff pressure of 30 cmH_2_O did not significantly alter the incidence of airway injury. However, the degree of tracheal mucosal damage significantly increased over time, with the 6, 8, and 10 h cohorts showing significantly higher injury levels than the 2 h cohort (*p* < 0.05). A similar trend was observed in the postoperative sore throat severity among different cohorts (Figures 4A–C). The degree of injury was positively correlated with postoperative sore throat severity (*r* = 0.6232, 95% CI of *r*: 0.4863 to 0.7303, *p* < 0.0001) (Figure 4D).

**Table 1:** The basic characteristics of patients.

Characteristics	2 h (*n* = 20)	4 h (*n* = 20)	6 h (*n* = 20)	8 h (*n* = 20)	10 h (*n* = 20)
Age (years)	50 ± 12.3	51 ± 11.2	48 ± 12.9	51 ± 11.3	49 ± 13.1
Male/Female	8/12	7/13	8/12	9/11	10/10
Height (cm)	162.9 ± 7.6	163.3 ± 7.2	164.2 ± 6.4	163.4 ± 7.0	162.5 ± 7.8
Weight (kg)	60.28 ± 9.1	61.52 ± 6.9	65.32 ± 10.4	60.44 ± 8.2	58.56 ± 8.5
BMI (kg/m^2^)	22.96 ± 3.39	23.26 ± 2.7	24.17 ± 4.01	22.73 ± 2.84	22.27 ± 3.17
Tracheal tube ID *n* (%)					
6.5#	2 (10)	1 (5)	3 (15)	2 (10)	4 (20)
7.0#	10 (50)	11 (55)	10 (50)	11 (55)	8 (40)
7.5#	8 (40)	8 (40)	7 (35)	7 (35)	8 (40)

Values are mean (SD) or number (proportion).

**Figure 5:**
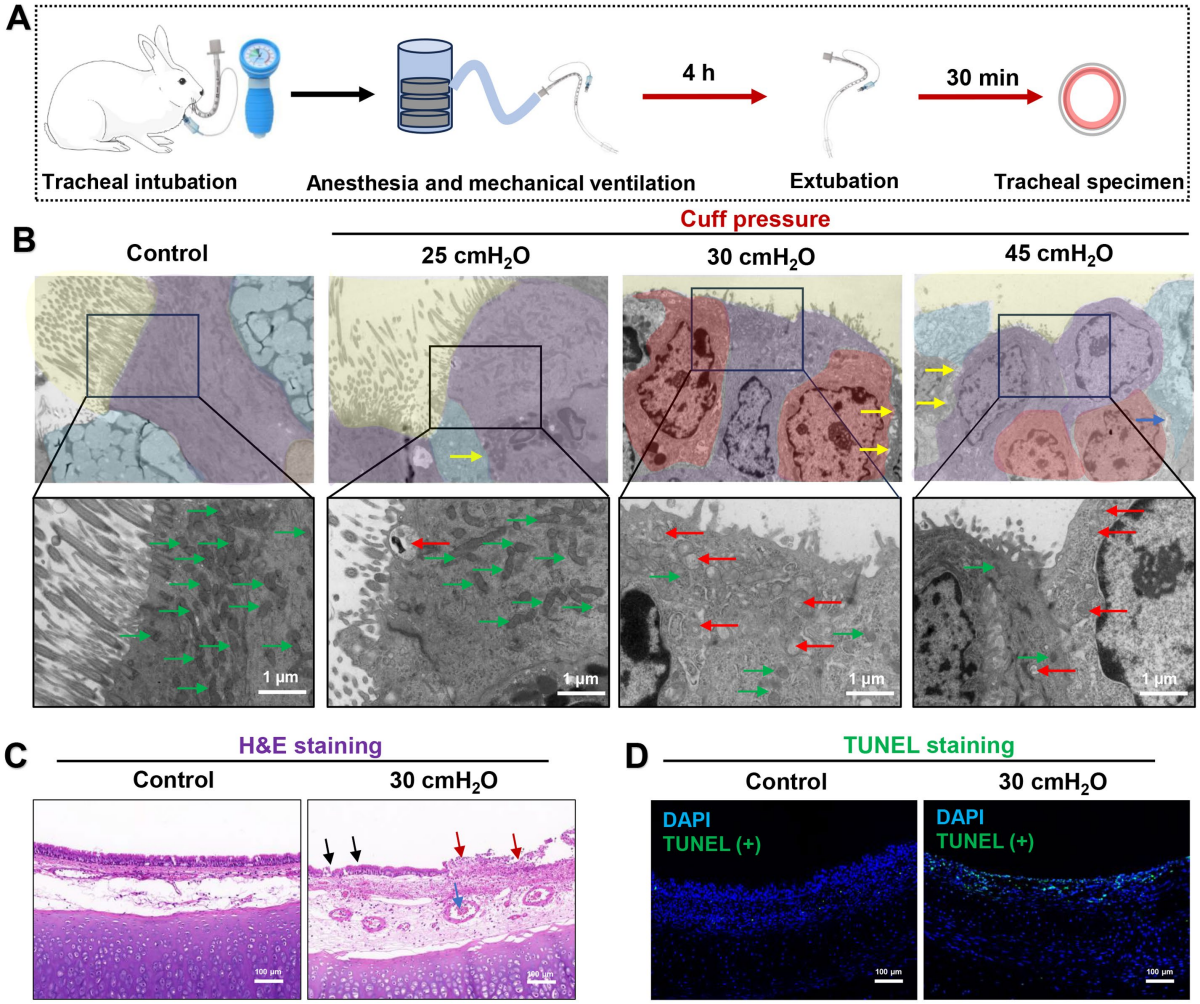
Effect of ETT cuff pressure on airway mucosa in a rabbit model of tracheal intubation mechanically ventilated. **(A)** Rabbit trachea intubation ventilation model flow chart. **(B)** Typical images of TEM microstructure of airway mucosa under different ETT cuff pressures. Airway cilia loss and inflammatory cell infiltration increased with increasing pressure. Ciliary structure of airway mucosa is shown in yellow background, fibrous columnar epithelium is shown in purple color, cup-shaped secretory cells in blue color, and inflammatory cells in red color. Yellow arrows indicate damage to the tight connections between cells, green arrows indicate normal mitochondria, and red arrows indicate damaged mitochondria, scale bar = 1 μm. **(C)** Typical H&E images of airway mucosa under 30 cmH_2_O ETT cuff pressure. The normal airway mucosal epithelium is continuous and complete, and the blood vessels are normal without obvious congestion. The airway mucosal damage, inflammatory exudation and blood vessels are obvious congestion caused by ETT cuff compression. Black arrows indicate disruption of airway cilia continuity, and red arrows indicate inflammatory exudation after mucosal injury. The blue arrow indicates microvascular thrombosis, scale bar = 100 μm. **(D)** Typical TUNEL staining images of airway mucosa under 30 cmH_2_O ETT cuff pressure; green fluorescence indicates apoptosis, the green fluorescence intensity represents the more severe the apoptosis of airway mucosal cells and blue fluorescence indicates DAPI, scale bar = 100 μm.

**Figure 6:**
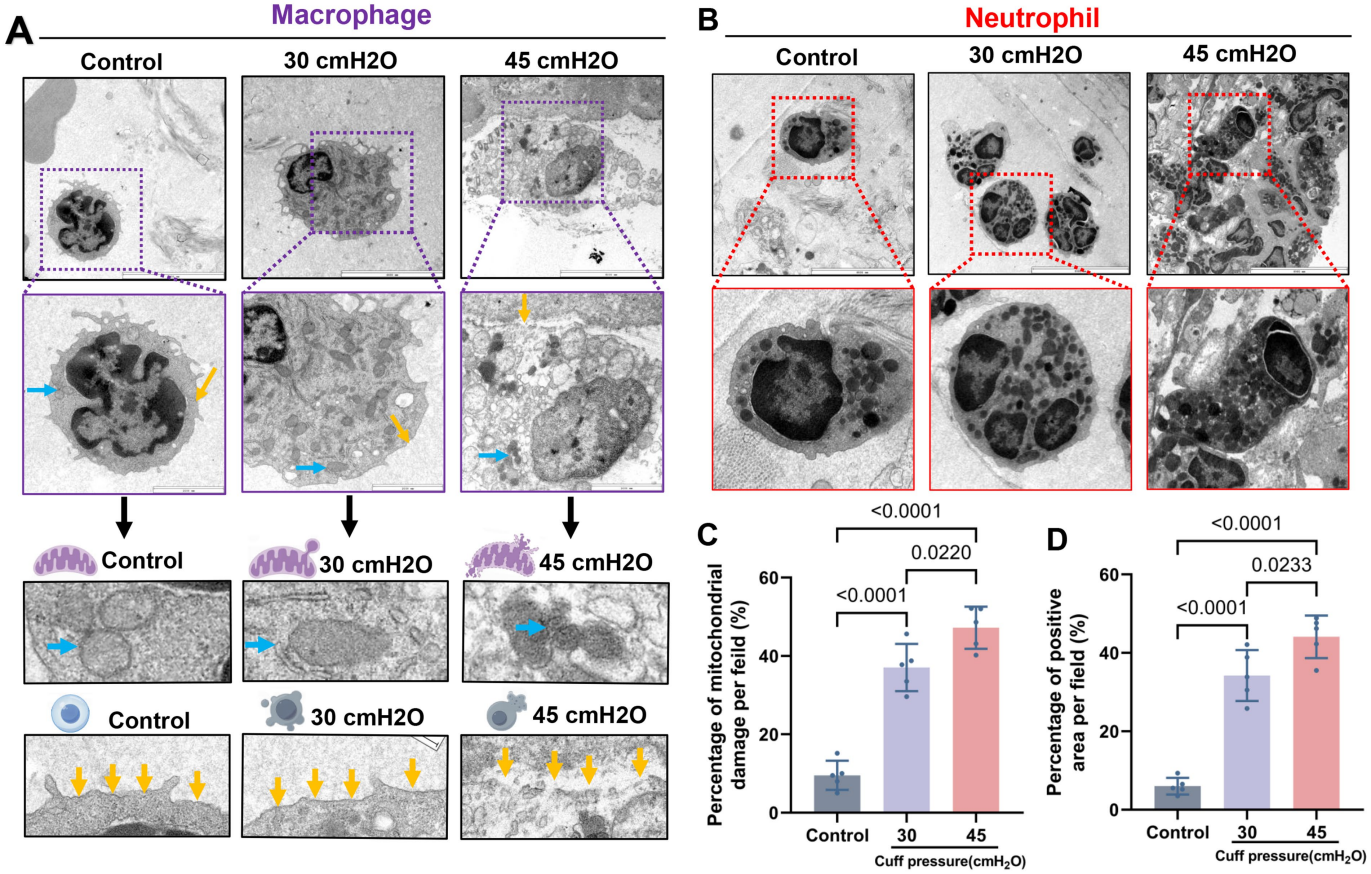
Microscopic structure of infiltrated inflammatory cells after mucosal injury. **(A)** Macrophages are typically altered, with the increase of pressure, the macrophage cell body expanded, the expression of lysosomes increased, and the mitochondrial swelling was obvious. With blue arrows indicating mitochondria and yellow arrows indicating cell membranes, scale bar = 2 μm. **(B)** Typical images of neutrophils, scale bar = 2 μm. **(C)** Proportion of mitochondrial damage in macrophages, data were shown as mean ± standard deviation. **(D)** Proportion of macrophage cell membrane breakage, data were shown as mean ± standard deviation.

TEM results of a rabbit mechanical ventilation model showed that a cuff pressure of 30 cmH_2_O significantly damaged the airway mucosal ciliated columnar epithelial cells (*n* = 5). This damage was characterized by substantial loss of cilia, mitochondrial swelling, and the occurrence of partial autophagy. A mechanical ventilation duration of 4 h at a cuff pressure of 30 cmH_2_O was sufficient to cause loss of airway mucosal epithelial cells and the tight connections between cells are broken (Figures 5A,B). H&E staining indicated that the pathological changes caused by a cuff pressure of 30 cmH_2_O primarily consisted of significant epithelial congestion, continuity changes, accompanied by partial disruption and exudative injury (Figure 5C). Beyond mechanical-induced changes, TUNEL staining suggested that a cuff pressure of 30 cmH_2_O could induce apoptotic changes in airway mucosal epithelial cells (Figure 5D). These findings suggest that maintaining cuff pressure at 30 cmH_2_O may not be a safe limit to prevent airway mucosal damage in patients undergoing prolonged mechanical ventilation. In addition to the changes in airway mucosal epithelial cells damage, we observed that cuff pressures of 30 cmH_2_O and 45 cmH_2_O led to inflammatory cell infiltration, predominantly consisting of macrophages and neutrophils. At a pressure of 30 cmH_2_O, notable damage to the mitochondria of macrophages was detected, characterized by mitochondrial swelling, reduced cristae, and partial disruption of the cell membrane. However, similar changes were not observed in neutrophils under the same cuff pressure of 30 cmH_2_O (Figure 6).

**Figure 7:**
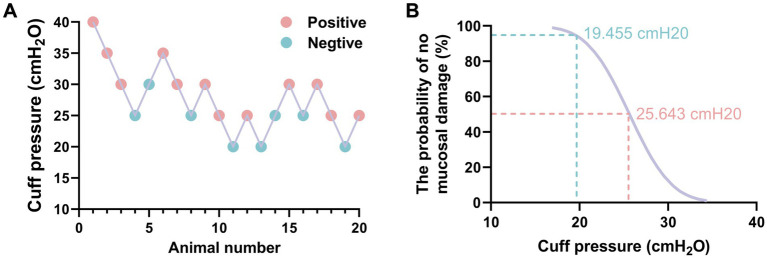
The minimum ETT cuff pressure for preventing airway mucosal injury was measured by sequential method. **(A)** Sequential chart of airway mucosa pressure values to prevent ETT cuff injury (*n* = 20). **(B)** Dose-response curves of ETT cuff pressure and probability of preventing airway mucosal damage.

A total of 20 adult rabbits were utilized in a sequential method to determine the minimum cuff pressure required to prevent airway mucosal damage (Figure 7A). Probit regression analysis revealed that the ED_50_ for preventing airway mucosal injury was 25.64 cmH_2_O (95% CI: 19.268–29.367 cmH_2_O), while the ED95 was 19.455 cmH_2_O (95% CI: 17.95–23.31 cmH_2_O) (Figure 7B). The validity of this injury probability prediction was confirmed by Pearson’s goodness-of-fit test (*χ*^2^ = 0.482, *p* = 0.923).

## Discussion

This study reevaluated the safety threshold of 30 cmH_2_O for ETT cuff pressure. Our findings, based on a retrospective clinical analysis and mechanical ventilation animal models, suggest that a cuff pressure of 30 cmH_2_O may be safe for airway mucosa during short-term mechanical ventilation. However, during prolonged mechanical ventilation, such pressures are likely to induce significant damage to the tracheal mucosal epithelium. Therefore, the traditionally recommended cuff pressure of 30 cmH_2_O may not effectively prevent tracheal mucosal injury. Furthermore, our survey indicated that anesthesiologists frequently exceed this recommended safety limit in practice. Moreover, various perioperative maneuvers can further elevate cuff pressure, compounding the risk of mucosal damage. These findings highlight the necessity of reassessing current guidelines and implementing more precise pressure monitoring to mitigate risks associated with extended mechanical ventilation.

The airway mucosa beneath the cuff is extremely fragile, and excessive cuff pressure can rapidly lead to severe mucosal injury (22). Unfortunately, the current situation is concerning. Our survey suggests that monitoring cuff pressure may not be a priority for anesthesiologists, who often rely on the imprecise finger touch method to inflate the cuff, resulting in pressures averaging 57.19 ± 30.99 cmH_2_O. In emergency surgeries and pre-hospital settings, higher cuff pressures are commonly used to prevent aspiration, making excessive initial cuff pressures a significant risk factor for mucosal injury (23). Furthermore, post-intubation changes in cuff pressure are critical. Our findings, consistent with prior studies, indicate that various intraoperative factors can increase cuff pressure, with coughing exerting the most significant impact. Nseir et al. (24) reported that about 73% of ICU patients experienced cuff over-inflation, highlighting the risks in scenarios without neuromuscular blocking agents, where coughing can severely damage the airways. Therefore, continuous monitoring and adjustment of cuff pressure are essential to prevent airway mucosal injury. A critical issue that demands attention is identifying the optimal safety threshold for cuff pressure. To balance effective airway sealing and prevention of mucosal damage, a commonly recommended safe limit remains at 30 cmH_2_O. This threshold, however, may need reevaluation in light of our findings. Unfortunately, no definitive guidelines on ETT cuff pressure have been published so far. Our findings may provide some reference for the development of clinical guidelines.

The rationale for setting a cuff pressure limit of 30 cmH_2_O as safe is based on the fact that the perfusion pressure of airway mucosal capillaries is approximately 35 cmH_2_O, suggesting that pressures within this limit should not impair mucosal blood flow (5, 6). However, this guideline fails to account for the perioperative period, when significant reductions in systemic blood pressure can inadvertently decrease mucosal perfusion pressure in anesthetized patients, potentially causing a 30 cmH_2_O cuff pressure to obstruct mucosal blood flow (25). Moreover, the venous hydrostatic pressure of the tracheal mucosa is about 24 cmH_2_O, and lymphatic return pressure is only 7 cmH_2_O (3). This disparity may explain the more pronounced damage caused by a cuff pressure of 30 cmH_2_O in patients requiring prolonged ventilation compared to those with shorter durations. There are no studies yet on the effects of a 30 cmH_2_O cuff pressure on the microscopic structure of the airway mucosa. Our observations suggest that significant mucosal injury can occur at this pressure during extended mechanical ventilation, indicating a need to reevaluate the safety limits of cuff pressure. It should be noted that airway mucosal injury caused by ETT cuff may be more serious in elderly patients, who are often complicated with airway inflammation and weaker cardiovascular function, which may lead to airway mucosal compression injury in elderly patients with ETT cuff replacement.

Based on animal models, we infer that the minimal cuff pressure to prevent airway mucosal injury is approximately 19.455 cmH_2_O. However, securing an airtight seal is also critical, as studies show that cuff pressures below 20 cmH_2_O increase the incidence of VAP by 2.5 times, making sub-20 cmH_2_O pressures a risk factor for VAP (26). The challenge of reducing cuff pressure while maintaining seal integrity necessitates exploring protective methods for the mucosa. Inflating the ETT cuff with an alkalinized lidocaine solution has been suggested to mitigate pressure fluctuations effectively (27, 28). Additionally, intraoperative cuff pressure adjustments and repositioning can protect the airway mucosa (1, 29). Our research indicates that mucosal injury at 30 cmH_2_O may involve inflammatory infiltration and cellular apoptosis. Therefore, developing drugs to enhance epithelial cell apoptosis tolerance could be crucial for protecting the airway mucosa. In addition, on the basis of our study, we would recommend continuous cuff pressure monitoring in patients undergoing prolonged mechanical ventilation, using an electronic manometer or arterial pressure sensor for continuous measurement, and adjusting the cuff pressure to less than 30 cmH_2_O as often as possible, according to titration to prevent air leakage.

In our study, we observed that during prolonged mechanical ventilation, ETT cuff compression caused inflammatory cell infiltration. Tracheal mucosal damage caused by ETT cuff is essentially a type of ischemia-reperfusion injury, stemming from an interruption in the respiratory chain that leads to an accumulation of reactive oxygen species (ROS), resulting in cellular pyroptosis under oxidative stress. Sustained oxidative stress contributes to the onset and progression of various diseases, with ROS production being a critical step in NLRP3-mediated pyroptosis (30–32). Our recent research has identified that NLRP3 activation-mediated pyroptosis of tracheal mucosal cells was a primary cause of ETT cuff-related mucosal damage (33). Therefore, in view of the activation of airway mucosal inflammation caused by ETT, reducing ROS is the key to prevent airway damage. Using reducing substances to neutralize ROS or developing new ETT cuff materials and nanocoatings to reduce inflammatory activation may be a new direction for airway protection. Our previous study also demonstrated that the natural reducing agent hydrogen (H_2_) was effective in reducing airway inflammation.

This study has several limitations. Firstly, our findings are based on data from selected areas within Sichuan Province, China, and did not utilize a rigorous stratified random sampling method. Secondly, strict exclusion criteria were formulated in the retrospective analysis, and a large proportion of patients were excluded, which may have excluded some important groups for the results of this study. The excluded population may have caused imbalance of potential influencing factors in different groups. Further expansion of the sample size for the retrospective analysis may be necessary. Additionally, our conclusion that a 30 cmH_2_O cuff pressure may be unsafe for patients undergoing prolonged ventilation stems from a retrospective study, which may be influenced by uncontrolled variables affecting result reliability and lacks long-term follow-up data. A rigorous prospective randomized controlled trial is needed to further validate these findings. Finally, the pathological results of this study were based on the animal model, and the animal model cannot fully simulate the human body. It is possible that the detection of human samples can further increase the reliability of the results and the basis for clinical application.

## Conclusion

Our study discovered a concerning level of attention that anesthesiologists pay to cuff pressure. Clinical retrospective analysis and animal models have linked the currently established cuff pressure of 30 cmH_2_O to airway mucosal damage in patients on prolonged mechanical ventilation. Considering these findings, it may be necessary to lower the safety threshold for cuff pressures and enhance educational efforts on cuff pressure management among anesthesiologists to reduce the risk of mucosal injury, however, the occurrence of reflux and aspiration should be prevented according to the actual situation.

## Data Availability

The raw data supporting the conclusions of this article will be made available by the authors, without undue reservation.
