# Supplementary material for: Reevaluating 30 cmH2O endotracheal tube cuff pressure: risks of airway mucosal damage during prolonged mechanical ventilation
Source: Front Med (Lausanne). 2024 Nov 25;11:1468310. doi: 10.3389/fmed.2024.1468310 (PMC11625575; doi:10.3389/fmed.2024.1468310)
Supplement: Supplementary file 1 [file Data_Sheet_1.DOCX]

**Supplementary Material 1**

**A questionnaire endotracheal tube air pressure**

First question: **Your hospital is** [single topic selection]

| **Option** | **Subtotals** | **Proportions** |
| --- | --- | --- |
| Triple A | 487 | 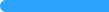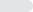76.81% |
| 3 B | 80 | 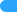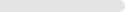12.62% |
| Second A | 36 | 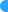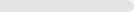5.68% |
| Below 2nd grade | 31 | 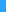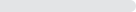4.89% |
| This question is valid for the number of people | 634 |  |

Question 2: **Your length of service** [single topic selection]

| **Option** | **Subtotals** | **Proportions** |
| --- | --- | --- |
| Within five years, | 237 | 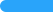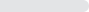37.38% |
| 5-10 years | 136 | 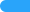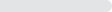21.45% |
| 10-20 years | 179 | 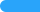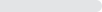28.23% |
| More than 20 years | 82 | 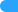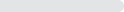12.93% |
| This question is valid for the number of people | 634 |  |

Questions 3: **Your title** [single topic selection]

| **Options** | **Subtotals** | **Proportions** |
| --- | --- | --- |
| Internship | 46 | 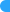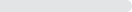7.26% |
| Resident | 210 | 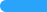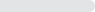33.12% |
| Attending physician | 243 | 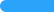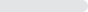38.33% |
| Deputy director of the physician and above | 135 | 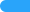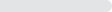21.29% |
| This question is valid for the number of people | 634 |  |

Questions 4: **You think the doctor anesthesia monitoring the endotracheal tube air pressure is importan**t [single topic selection]

| **Options** | **Subtotals** | **Proportions** |
| --- | --- | --- |
| It is very important | 304 | 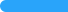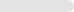47.95% |
| Important | 274 | 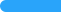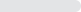43.22% |
| General | 55 | 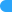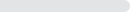8.68% |
| Doesn't matter | 1 | 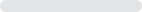0.16% |
| This question is valid for the number of people | 634 |  |

Questions 5: **You have been monitoring the endotracheal tube air pressure knowledge guidance and training** [single topic selection]

| **Options** | **Subtotals** | **Proportions** |
| --- | --- | --- |
| Have had multiple | 79 | 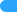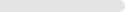12.46% |
| Yes, but rarely | 310 | 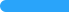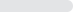48.9% |
| There was a | 30 | 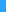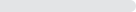4.73% |
| There is no | 215 | 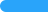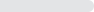33.91% |
| This question is valid for the number of people | 634 |  |

Question 6: **Does your hospital have endotracheal tube cuff pressure monitoring equipment?** [Single choice]

| **Options** | **Subtotals** | **Proportions** |
| --- | --- | --- |
| is | 98 | 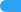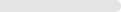15.46% |
| no | 536 | 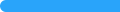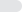84.54% |
| This question is valid for the number of people | 634 |  |

Question 7: **You use a pressure gauge on the endotracheal tube air pressure monitoring** [single topic selection]

| **Options** | **Subtotals** | **Proportions** |
| --- | --- | --- |
| Conventional detection | 31 | 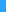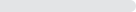4.89% |
| Frequent monitoring | 14 | 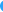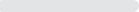2.21% |
| Occasional monitoring | 86 | 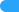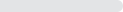13.56% |
| Never monitoring | 503 | 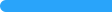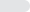79.34% |
| This question is valid for the number of people | 634 |  |

Question 8: **The topic you think reasonable air pressure should be how much** [single topic selection]

| **Options** | **Subtotals** | **Proportions** |
| --- | --- | --- |
| 10-20 cmH_2_O | 282 | 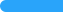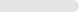44.48% |
| 20-30 cmH_2_O | 253 | 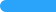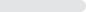39.91% |
| 30-40 cmH_2_O | 17 | 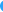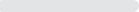2.68% |
| More than 40 cmH_2_O | 2 | 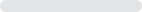0.32% |
| Don't know | 80 | 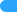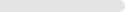12.62% |
| This question is valid for the number of people | 634 |  |

Question 9: **How do you usually give endotracheal tube balloon gas injection?** [Single choice]

| **Options** | **subtotal** | **Proportions** |
| --- | --- | --- |
| Fill with gas at a fixed volume | 37 | 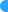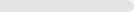5.84% |
| Feel with your fingers manometry (touches such as tip) | 526 | 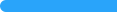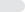82.97% |
| Measure your pressure according to a pressure gauge | 11 | 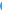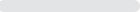1.74% |
| By auscultation not leak into the smallest volume | 60 | 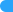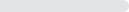9.46% |
| This question is valid for the number of people | 634 |  |

Question 10: **If the operation time is longer, do you think it is necessary to monitor the cuff pressure repeatedly?** [single topic selection]

| **Options** | **Subtotals** | **Proportions** |
| --- | --- | --- |
| Need to be | 609 | 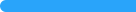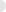96.06% |
| Don't need | 25 | 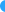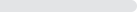3.94% |
| This question is valid for the number of people | 634 |  |

Question 11: **If the operation for a long time, how many reasonable gasbag pressure monitoring frequency interval time? (if you think you need to repeat monitoring** [single topic selection]

| **Option** | **Subtotals** | **Proportions** |
| --- | --- | --- |
| 2 h | 404 | 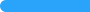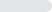63.72% |
| 4 h | 159 | 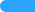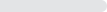25.08% |
| 8 h | 6 | 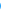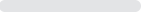0.95% |
| Have not considered | 30 | 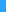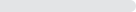4.73% |
| (Empty) | 35 | 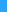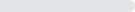5.52% |
| This question is valid for the number of people | 634 |  |

Question 12. **If the operation time is long, do you think it is necessary to deflating the cuff pressure regularly?** [Single choice questions]

| **Options** | **Subtotals** | **Proportions** |
| --- | --- | --- |
| Need | 597 | 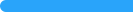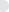94.16% |
| Not needed | 37 | 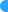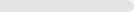5.84% |
| This question is valid for the number of people | 634 |  |
